# Multistep Ion Channel Remodeling and Lethal Arrhythmia Precede Heart Failure in a Mouse Model of Inherited Dilated Cardiomyopathy

**DOI:** 10.1371/journal.pone.0035353

**Published:** 2012-04-13

**Authors:** Takeshi Suzuki, Takao Shioya, Takashi Murayama, Masami Sugihara, Fuminori Odagiri, Yuji Nakazato, Hiroto Nishizawa, Akihito Chugun, Takashi Sakurai, Hiroyuki Daida, Sachio Morimoto, Nagomi Kurebayashi

**Affiliations:** 1 Department of Cellular and Molecular Pharmacology, Juntendo University Graduate School of Medicine, Bunkyo-ku, Tokyo, Japan; 2 Department of Cardiovascular Medicine, Juntendo University Graduate School of Medicine, Bunkyo-ku, Tokyo, Japan; 3 Department of Physiology, Faculty of Medicine, Saga University, Saga, Japan; 4 Department of Clinical Pharmacology, Faculty of Medical Sciences, Kyushu University, Fukuoka, Japan; Centro Cardiologico Monzino, Italy

## Abstract

**Background:**

Patients with inherited dilated cardiomyopathy (DCM) frequently die with severe heart failure (HF) or die suddenly with arrhythmias, although these symptoms are not always observed at birth. It remains unclear how and when HF and arrhythmogenic changes develop in these DCM mutation carriers. In order to address this issue, properties of the myocardium and underlying gene expressions were studied using a knock-in mouse model of human inherited DCM caused by a deletion mutation ΔK210 in cardiac troponinT.

**Methodology/Principal Findings:**

By 1 month, DCM mice had already enlarged hearts, but showed no symptoms of HF and a much lower mortality than at 2 months or later. At around 2 months, some would die suddenly with no clear symptoms of HF, whereas at 3 months, many of the survivors showed evident symptoms of HF. In isolated left ventricular myocardium (LV) from 2 month-mice, spontaneous activity frequently occurred and action potential duration (APD) was prolonged. Transient outward (*I_to_*) and ultrarapid delayed rectifier K^+^ (*I_Kur_*) currents were significantly reduced in DCM myocytes. Correspondingly, down-regulation of Kv4.2, Kv1.5 and KChIP2 was evident in mRNA and protein levels. In LVs at 3-months, more frequent spontaneous activity, greater prolongation of APD and further down-regulation in above K^+^ channels were observed. At 1 month, in contrast, infrequent spontaneous activity and down-regulation of Kv4.2, but not Kv1.5 or KChIP2, were observed.

**Conclusions/Significance:**

Our results suggest that at least three steps of electrical remodeling occur in the hearts of DCM model mice, and that the combined down-regulation of Kv4.2, Kv1.5 and KChIP2 prior to the onset of HF may play an important role in the premature sudden death in this DCM model. DCM mice at 1 month or before, on the contrary, are associated with low risk of death in spite of inborn disorder and enlarged heart.

## Introduction

Inherited dilated cardiomyopathy (DCM) is a progressive disease characterized by an enlarged and weakened heart. It has recently become clear that gene mutations in various cytoskeletal and sarcomeric proteins that lead to weakness in the systems involved in force production can contribute to the development of DCM [1]–[6]. The cause of death among patients with DCM is severe heart failure (HF) (∼50%), where cardiac output is substantially decreased. In addition, lethal arrhythmias often occur before symptoms of HF are evident, resulting in sudden death (SD) (30–40%) [2], [7].

At present, it remains unclear how HF and arrhythmogenic changes proceed in carriers of DCM mutations. In many cases, they are asymptomatic until symptoms of HF, such as breathlessness or tiredness, manifest [2], despite the inborn weakness in force production, indicating that their hearts are compensated until some event drives them to HF. In addition, their myocardium might be arrhythmogenic before or during development of HF, although clear evidence is still lacking. In humans, systematic investigation of the functional properties of the DCM heart has been difficult because the data are confounded by many background factors such as complication from other diseases, therapeutic intervention, environmental conditions, genetic factors, etc. [8].

A variety of genetically modified mouse models that develop characteristics of DCM have been created in recent years by knockout, knockdown or overexpression of some specific genes [9]–[15]. These models show variable phenotypes of arrhythmia, i.e., abnormal conduction, bradycardia, ventricular tachycardia and ventricular fibrillation, long QT syndrome, or no reports of arrhythmia, depending on their genetic modifications [9]–[17]. Although these models are potentially useful for investigation of DCM, it is unclear which type of familial DCM they mimic, because most of their genetic modifications are not seen in familial DCM. To understand the mechanisms of arrhythmogenesis in familial DCM, investigations with knock-in animal models based on real human DCM mutation are particularly needed [14], [17].

Mutations in the cardiac troponin T gene (*TNNT2*) are reported in ∼3% of DCM patients [3], [6], [18]. Among them, a deletion mutation of K210 (ΔK210, also known as ΔK217) that decreases Ca^2+^ sensitivity in force development accounts for one-third of all mutations found in *TNNT2* [3], [6], [18]. This mutation is believed to be a recurrent mutation in human DCM, probably because the site is susceptible to deletion mutation [18]. Onset of HF symptoms or occurrence of SD in these DCM patients with the ΔK210 mutation is variable, ranging from 10 to 70 years of age.

Recently, a knock-in mouse model of DCM caused by the ΔK210 mutation was created based on the human familial DCM [14], [19]. The mutant mice showed markedly enlarged hearts and significantly lower Ca^2+^ sensitivity in force generation than wild-type (WT) mice. The developed force of acutely isolated papillary muscles from the mutant and WT mice was similar because the peak amplitude of the Ca^2+^ transients in cardiomyocytes was increased in mutant mice, suggesting that the decreased contractility was compensated by an increase in the Ca^2+^ transients [14]. In many cases, these mice appeared to die suddenly with a t_1/2_ of 70 days. Telemetric ECG recording showed Torsades de Pointes at their SD [14], suggesting that arrhythmogenic changes may occur. This model thus appears to resemble the phenotypes of human DCM [14], [20]–[21].

The aim of this study was to clarify (1) when HF develops, and (2) whether, and if so how, electrical remodeling occurs in the DCM model mice with the ΔK210 mutation. We assessed spontaneous activity in myocardium, action potential (AP) configuration and ionic currents in isolated myocytes. In addition, we carried out real-time RT-PCR and Western blot analysis for ion channels and associated proteins potentially involved in the functional changes seen in DCM. Our results indicate that multiple types of progressive electrical remodeling occur at different time points in the hearts of DCM model mice. Clear symptoms of congestive HF are preceded by this electrical remodeling.

## Results

### In vivo properties of DCM model mice at 1, 2 and 3 months

**Figure 1:**
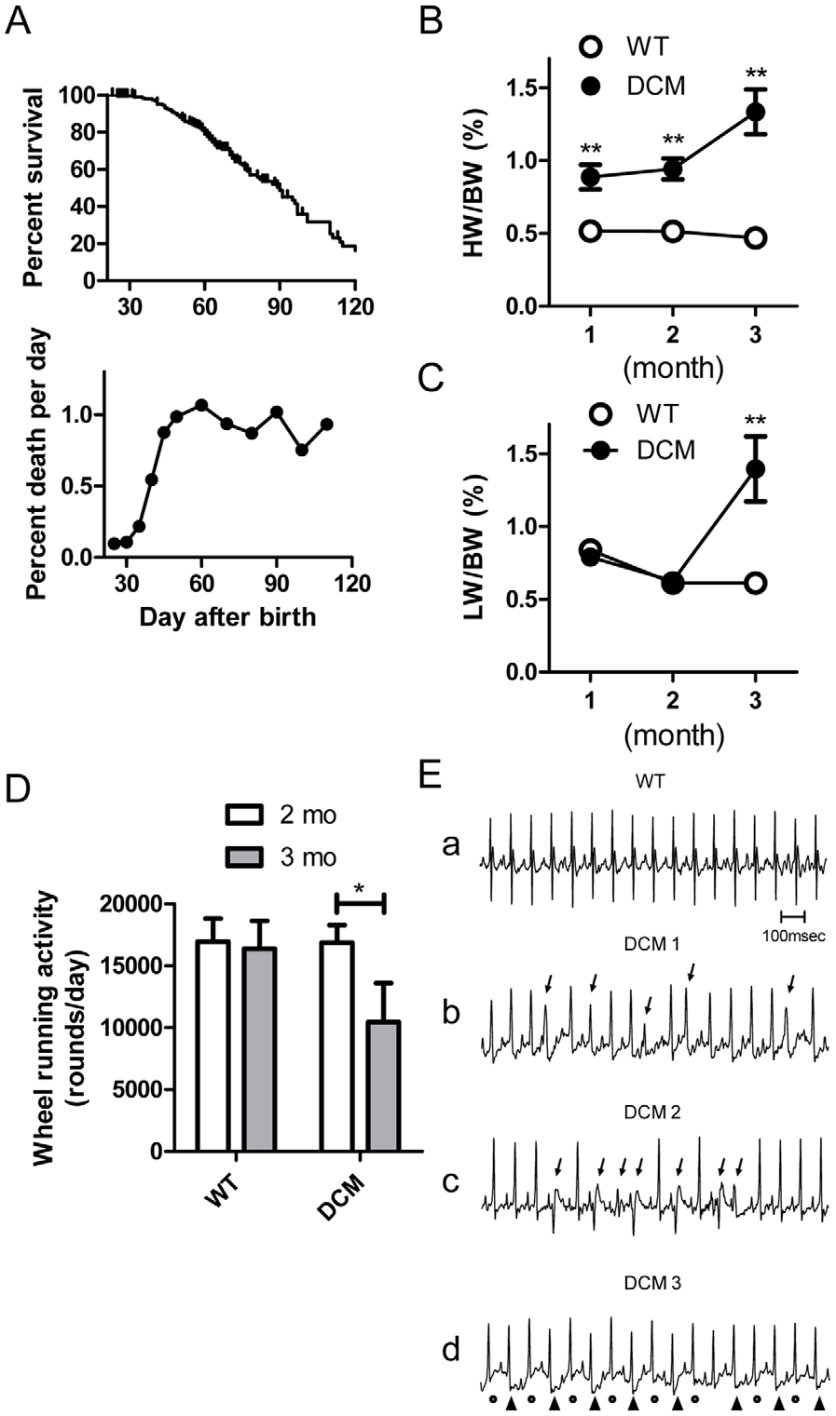
In vivo data of DCM mice. **A**. Kaplan-Meier survival curves for DCM mice (n = 211) and their % death in our laboratory. **B & C**. HW/BW ratio (**B**) and LW/BW ratio (**C**) in WT (n = 5–7) and DCM mice (n = 7 for 1 and 2 months; n = 14 for 3 months). **P<0.01 between WT and DCM. **D**. Average wheel running activity of WT and DCM mice at 2 and 3 months. Data at 2 and 3 months were obtained from the same animals (n = 7). *P<0.05 between 2 and 3 months. **E**. A typical normal ECG trace in WT (a) and representative traces of PVCs (b, c) and T-wave alternans (d) obtained in conscious DCM mice at 2 months. Arrows in b and c indicate premature ventricular contractions (PVCs) occurred before atrioventricular conduction. Dots and arrowheads in d indicate beat-to-beat alteration in T-waves.

Because the mortality of DCM model mice abruptly changes at around 1.5 months of age (Fig. 1A) [14], we compared in vivo properties of DCM mice at 1, 2 and 3 month. DCM mice had significantly enlarged hearts with average heart weight/body weight (HW/BW) ratio of about twice the WT mice at 1 and 2 months (Fig. 1B) [14] and even at birth (WT: 0.60±0.03% (n = 7); DCM: 0.97±0.05% (n = 9)). The enlargement became more prominent at 3 months. To determine when HF develops in DCM mice, lung weight/body weight (LW/BW) ratio and voluntary exercise activity were determined because HF is defined as inability of the heart to supply sufficient blood flow to meet the body's needs and is accompanied by symptoms such as shortness of breath (dyspnea) and exercise intolerance. The LW/BW ratio, indicative of lung edema, was similar between DCM and WT mice until 2 months. In DCM mice that survived to 3 months of age, average LW/BW ratio significantly increased (Fig. 1C). Physical activity was measured using a voluntary running wheel [22]. At 2 months, wheel-running activity of DCM mice was comparable to WT mice. However, at 3 months, running activity was significantly decreased in DCM mice, whereas the activity in the WT remained constant (Fig. 1D). These data suggest that congestive HF and resultant pulmonary edema start to develop in DCM mice between 2 and 3 months.

Table 1 shows in vivo parameters of WT and DCM mice. There were no significant differences in body weights between WT and DCM mice during 1–3 months. ECG records were obtained from anesthetized and conscious mice. Although heart rate (HR) under anesthetized condition was significantly higher in DCM than WT mice at 1 month, this is probably due to difference in depth of anesthesia. In conscious mice, HR was similar between WT and DCM mice at the same age. ECG recording of 2-month mice showed prolongation of QRS and QT intervals in DCM mice as reported previously [14], [21], whereas the prolongations were less marked at 1 month (Table 1). Telemetric ECG recording of mice at 2 months showed Torsades des pointes at their death as reported previously (n = 3) [14]. Noninvasive ECG records were obtained for 30 min from conscious mice of 2 months by restricting free movement in an oval plastic dome with openings at mouth and tail (see methods). This procedure increased stress of mice to enhance propensity for arrhythmias and allowed us to analyze many mice repeatedly. Premature ventricular complexes (PVC) were recorded from 3 out of 6 DCM mice during the 30 min recording period, whereas none were recorded in WT (n = 5) (Fig. 1E). Alternating T-wave morphology (T-wave alternans) was observed in 3 out of 6 DCM mice (1 mouse showed both PVC and T wave alternans). PVC (3 mice) and T wave alternans (2 mice) were also obtained in DCM mice at 3 months (n = 5). In addition to the abnormality in ECG, DCM mice at 3 months became highly sensitive to anesthesia: with 3-month DCM mice, 4 out of 10 mice died from respiratory arrest during anesthesia (20 mg/kg pentobarbital injection); whereas 2-month mice or younger survived at this dose.

**Table 1:** Summary of in vivo parameters.

Age	1 month		2 months		3 months	
	WT	DCM	WT	DCM	WT	DCM
BW (g)	12.8±0.9	13.3±0.6	21.4±0.6	21.7±0.8	23.2±1.0	22.7±0.7
*N*	8	8	18	13	11	16
ECG recording (anesthetized)						
HR (bpm)	355±27	430±25* ^,^ a	392±18	394±37	ND	ND
PR (ms)	35±1	33±1	38±2	38±2	ND	ND
QRS (ms)	9.6±0.3	10.3±0.3	11.0±0.3	16.7±0.6*	ND	ND
QT (ms)	34±1	42±2*	36±1	53±1*	ND	ND
*N*	6	6	6	6	-	-
ECG recording (conscious)						
HR (bpm)	740±16	773±15	721±10	700±6	726±9	698±17
PR (ms)	29.1±0.9	29.6±0.8	33.6±0.7	33.0±1.1	34.7±0.8	33.8±1.6
QRS (ms)	9.9±0.4	12.3±0.4	10.1±0.4	15.9±0.9**	10.1±0.1	16.9±1.4**
QT (ms)	17.1±0.4	23.3±0.9**	17.0±0.5	33.8±1.9**	18.5±0.6	37.9±2.2**
*N*	5	6	5	6	5	5

Data are means ± SEM.

* P<0.05,

** P<0.01 vs. WT. BW: body weight. ND: not determined.

a : The significant difference from WT mice is probably due to a difference in depth of anesthesia.

### Ventricular spontaneous activity assessed by membrane potential imaging and force monitoring in isolated myocardium

**Figure 2:**
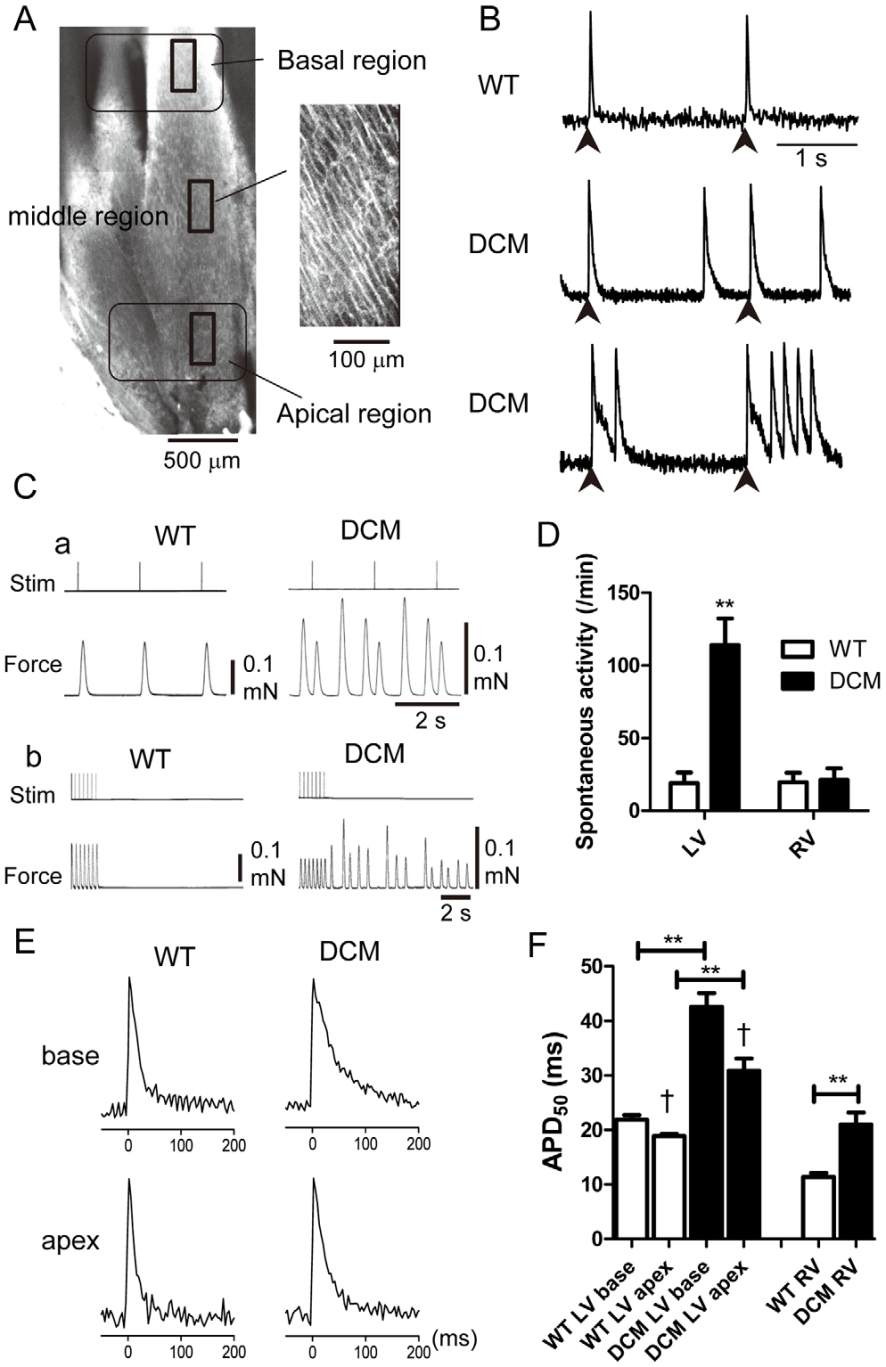
AP signals and force records obtained from WT and DCM LV at 2 months. **A**. Typical images of the endocardial surface of live LV muscle from a WT mouse. Left: The LV muscle wall observed from the endocardial side using a 4× objective. Six images were combined to produce the full image. Right: Image of cardiac surface muscle cells (256×512 pixels, 160×320 µm area) observed with a 20× objective. Action potential signals shown in B and E were obtained from this size of area. **B**. Representative AP signals recorded from the LVs of WT (top) and DCM (middle and bottom) mice stimulated at 0.5 Hz. AP signals were obtained from middle region as 8×16 pixel images at 3.67 ms intervals. Arrowheads indicate field stimulation. Experiments were carried out at 25–27°C. **C**. Representative traces showing force development in LV papillary muscle from a WT (left) and DCM (right) mouse during 0.5 Hz field stimulation (**a**) and during and after 3 Hz field stimulation (**b**). **D**. Average frequency of spontaneous contractions after 3 Hz field stimulation in LV and RV. Means ± SEM. (WT, n = 10; DCM, n = 14). **E**. Representative AP signals from the basal and apical regions of endocardial surface (see Panel A) of a LV stimulated at 0.5 Hz. **F**. Comparison of APD_50_ values in the basal and apical regions of LVs and center region of RVs from WT (LV: base, n = 41; apex, n = 41 from 10 hearts, RV center region: n = 18 from 4 hearts,) and DCM (base, n = 85; apex, n = 64 from 14 hearts, RV: n = 35 from 7 hearts). Means ± SEM. **P<0.01 between WT and DCM. †P<0.05 between base and apex in LV.

Since DCM model mice show a high incidence of death beginning around 1.5 months (Fig. 1A) [14], we initially monitored optical AP signals in isolated cardiac muscles from 2-month mice to determine what occurs in DCM myocardium (Fig. 2). The myocardium from 2-month WT and DCM hearts was loaded with di-4-ANEPPS, and AP signals were recorded from the endocardial side using a confocal microscope/W-view system as described previously [23] (Fig. 2A). In WT LV, AP signals were only elicited in response to stimulation, whereas DCM LV exhibited a large number of spontaneous APs (Fig. 2B). The DCM myocardium exhibited both sporadic APs (APs developing at rest) and burst-firing (APs developing during the repolarizing phase), in addition to the stimulus-induced ones.

Spontaneous activities were also confirmed as spontaneous contractions in papillary muscles from LV. The records in Fig. 2C-a show typical isometric contractions of LV papillary muscle elicited by stimulation at 0.5 Hz. The WT LV showed twitch contractions only upon stimulation, but frequent spontaneous contractions were observed between stimuli in the DCM LV. Fig. 2C-b shows force records collected during and after a period of stimulation at 3 Hz. The WT LV showed twitch contractions upon stimulation, and infrequently contracted after the stimulation was stopped. The DCM muscle also responded to the 3 Hz stimuli, but continued with frequent spontaneous contractions after the stimulation period ended. Spontaneous contractions were much more frequent in DCM LV (Fig. 2D). Similar measurements were carried out with right ventricle (RV) and left atrial (LA) muscles. Spontaneous activities were rarely observed in RV (Fig. 2D) and LA (not shown) from both WT and DCM mice. Myocardial automaticity appears to be considerably enhanced in LVs.

### Prolongation of AP duration (APD) in DCM LVs

Another notable change in the AP signals from DCM LVs was an obvious lengthening of APD (Fig. 2E). Because ventricular APD is known to be spatially different and is longer in the basal region than the apical region of endocardium [24], APD was measured in both regions in LV (Fig. 2F, left 4 columns). APD_50_ values (APD at 50% repolarization) were used because they are relatively stable compared to longer APD measurements (e.g., APD_80_); APD_80_ measurements are more variable as they depend on the amount of Na^+^-Ca^2+^ exchange (NCX) activity and they often suffer from movement artifacts. In the basal region of WT mice, the average APD_50_ was approximately 20 ms, which was comparable to previous reports obtained from Langendorf-perfused mouse heart using monophasic action potential recording [25]–[26]. APD_50_ in DCM LVs was about 2-fold longer than that in WT LVs. In the apical region, the APD_50_ from DCM mice was about 1.6-fold longer than that from WT mice. In DCM RV, APD_50_ taken from center region was also significantly prolonged in DCM compared to WT, however, the absolute value was much shorter than in LV (Fig. 2F, right 2 columns). Thus, APD was significantly prolonged in DCM ventricle with the spatial difference tending to be increased. The prolongation of APD is consistent with the prolonged QT interval in DCM mice (Table 1). In the following experiments, we mainly investigated properties of LVs from WT and DCM mice.

### AP and current recording by whole-cell clamp

**Figure 3:**
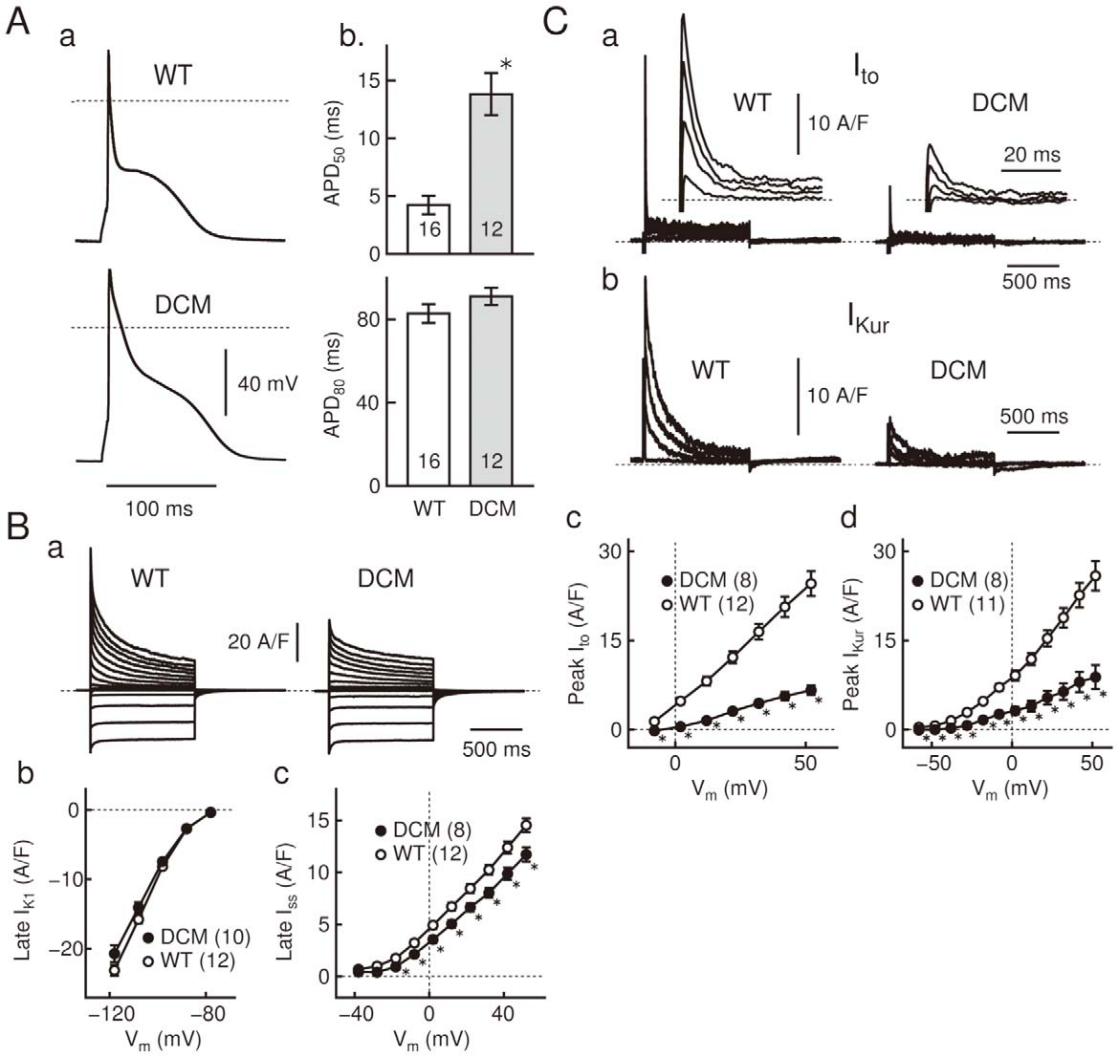
DCM-associated alterations in AP waveform and membrane currents. **A**: APs of whole-cell clamped WT and DCM cells (a), evoked at 5 Hz. The APD_50_ and APD_80_ (b) of WT (n = 16) and DCM cells (n = 12) were measured at repolarization to −18 and −58 mV, respectively. **B**: Records of whole-cell current from WT and DCM cells (a), activated by voltage-steps (1 s to −118 - +52 mV in 10 mV increment) from a holding potential of −78 mV. I–V relations of *I_K1_* (b) and *I_ss_* (c) in DCM (n = 10 or 8) and WT cells (n = 12) were measured as the steady-state amplitude of the whole-cell current at pulse end. **C**: *I_to_* (a) and *I_Kur_* (b) of WT and DCM cells, isolated using 100 µM-4AP and an inactivation prepulse (See Method S1). The records were acquired with depolarizing pulses to +52, +32, +12, and to −8 mV. Insets expand the peaks in a faster time-base. I–V relations of *I_to_* (c) and *I_Kur_* (d) compile their peak amplitudes in DCM (n = 8) and WT cells (n = 12 or 11). Dotted lines: zero-voltage or zero-current level. Data are means ± SEM. *p<0.05 between WT and DCM. Fig. 3B-a: Cm = 139 (WT) and 152 pF (DCM).

In Fig. 3, we conducted whole-cell clamp experiments to further examine the DCM-associated AP prolongation found in the preceding optical measurement. For this purpose, single LV cells from DCM and WT mice were whole-cell clamped in physiological conditions at 37°C. Current-clamped DCM cells had a resting membrane potential of −75.0±0.4 mV (n = 16), which was statistically indistinguishable from the value of WT cells (−76.1±0.5 mV, n = 12).

Electrical stimulation (5 Hz) evoked APs having a spike-and-dome configuration in both cells (Fig. 3A-a). The spike was much broader in DCM cells than in WT cells due to a reduced rate of early-phase repolarization; accordingly, the APD_50_ of DCM cells was ∼3 times as large as that of WT cells (Fig. 3A-b). Terminal repolarization of the AP was more variable in both WT and DCM; accordingly, the APD_80_ of DCM cells was 10% larger than in WT cells but without statistical significance. These findings on AP prolongation are consistent with the preceding optical measurements (Fig. 2B, E, F).

We then explored alterations in the membrane current systems that underlie the DCM-associated APD prolongation. For this purpose, the cells were voltage-clamped at a holding potential of −78 mV, and whole-cell current was activated by 1 s square pulses. As illustrated in Fig. 3B-a, the outward whole-cell current activated by depolarizing pulses had a smaller amplitude in DCM cells than in WT cells, supporting the slower AP repolarization observed in DCM cells. In contrast, the inward current activated by hyperpolarizing pulses, which is mostly attributable to the inward-rectifier K^+^-current (*I_K1_*), in DCM cells had a comparative amplitude to WT cells (Fig. 3B-b).

The outward whole-cell current consists of three components as was previously reported [27]. We therefore explored the DCM-associated alterations in each component: the transient outward K^+^-current (*I_to_*), the ultrarapid delayed rectifier K^+^-current (*I_Kur_*), and the slowly-activating sustained K^+^-current (*I_ss_*). The *I_ss_* in DCM cells (isolated as the steady-state whole-cell current at pulse end) had a smaller but comparable (∼90%) amplitude to WT cells (Fig. 3B-c); however, the *I_to_* and *I_Kur_* in DCM cells (see Method S1 for their isolation) had substantially smaller peak amplitudes than WT cells (∼30%) (Fig. 3C). Therefore, the smaller amplitude of the outward whole-cell current can be attributed to the reduction of *I_to_* and *I_Kur_* in DCM cells.

### Gene expression and Western blot analyses in LV

Since the above results revealed a functional decrease in ionic channel activities carrying *I_to_* and *I_Kur_*, we analyzed the expression levels of the genes encoding the various ion channels that carry *I_to_* (Kv4.2), *I_K1_* (Kir2.1 and Kir2.2), *I_ss_* (Kv2.1) and *I_Kur_* (Kv1.5) [28]–[29]. The genes encoding the accessory subunit KChIP2, which contributes to *I_to_*, and the homeodomain transcription factor Irx5, which negatively regulates expression of Kv4.2 were also determined [30]–[31]. In addition, Kir3.1 (K_ACh_ channel), Nav1.5 (voltage-gated Na^+^ channel), Cav1.2 (L-type Ca^2+^ channel), Cav3.1 (T-type Ca^2+^ channel) and NCX1 (Na^+^-Ca^2+^ exchanger) were determined because they also may affect resting membrane potential and AP configuration [28]–[29], [32]–[34].

**Figure 4:**
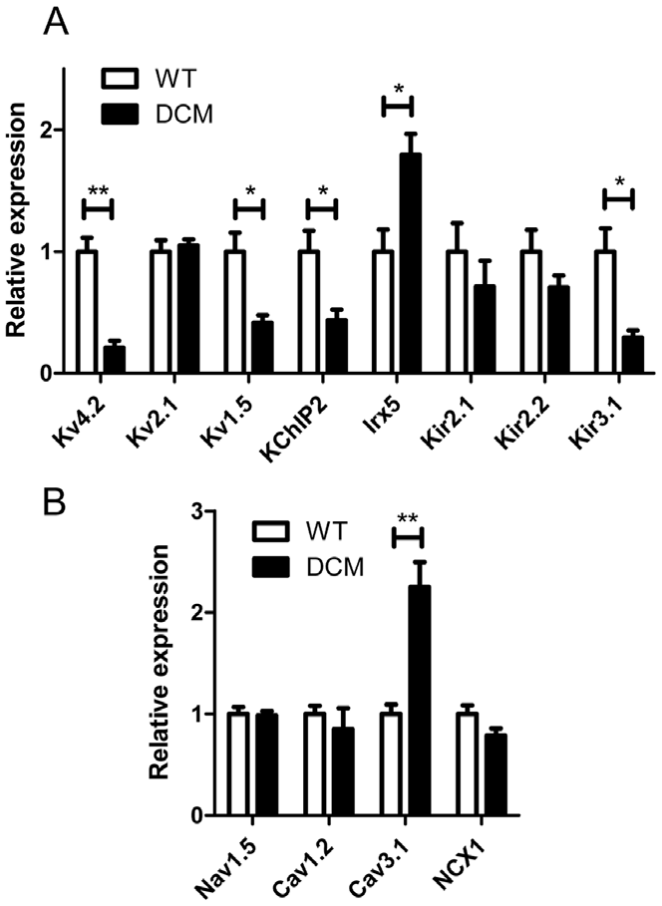
Relative expression levels of mRNA encoding the indicated ion channels and auxiliary subunits in LVs. Quantitative real-time PCR analysis was carried out with LVs of 2-month WT (n = 7) and DCM mice (n = 7). GAPDH gene was used as an internal control. Data from individual samples were normalized to the average of WT mice. **A**. Voltage-dependent K^+^ channels, inwardly rectifying K^+^ channels and related proteins. **B**. Voltage-dependent Na^+^ channels, Ca^2+^ channels and Na^+^-Ca^2+^ exchanger. Plots are means ± SEM (n = 7). **P<0.01, *P<0.05 between WT and DCM.

In DCM LVs, the expression levels of Kv4.2, KChIP2, Kv1.5 and Kir3.1 mRNAs were significantly diminished to about half or less of those in WT LVs (Fig. 4A). Conversely, the expression levels of Irx5 and Cav3.1 were increased 2-fold or more in DCM LVs (Fig. 4A and B). The increase in Irx5 is consistent with the down-regulation of Kv4.2 [31]. The expression levels of Kir2.1 and Kir2.2 were slightly lower in DCM LVs than WT LVs, though the difference was not significant. There was no significant difference in the expression level of Kv2.1, Cav1.2, Nav1.5 or NCX1 between WT and DCM LVs. There were also no significant changes in expression of Kir6.1 and Kir6.2, K_ATP_ channels (data not shown).

**Figure 5:**
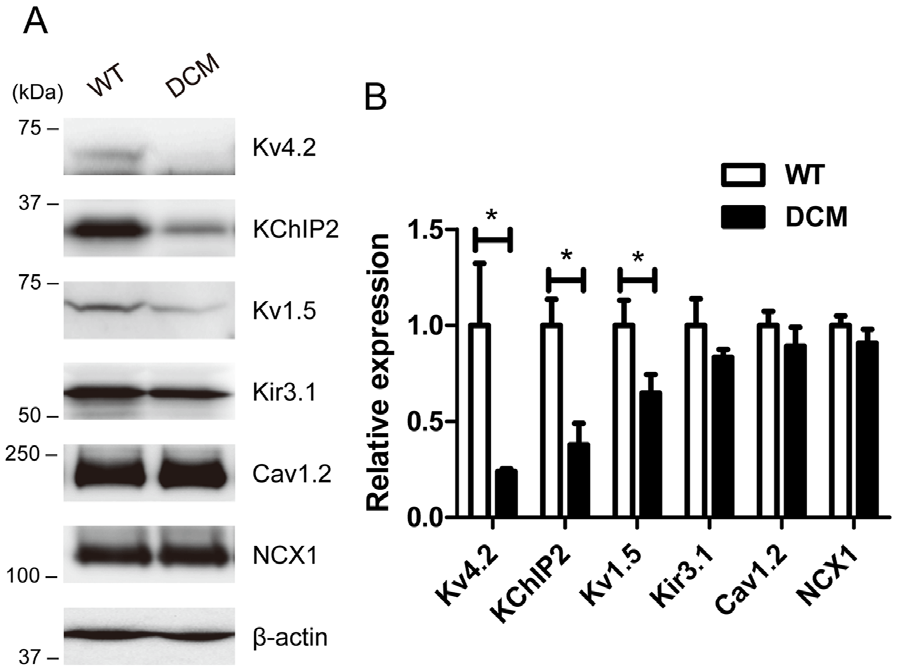
Western blot analysis of major channel proteins in LV. The membrane protein samples (50 µg protein for each) from 2-month WT (n = 4) and DCM LVs (n = 5) were separated by SDS-PAGE and Western blot analysis was carried out. See Methods for detail. **A**. Representative immunoblots of individual experiments. **B**. Averaged expression levels. The relative expression levels for each protein were normalized to the average value for the WT. Data are means ± SEM (WT: n = 4, DCM: n = 5). **P<0.01 between WT and DCM.

Expression of channel proteins, for which mRNA was decreased by two-fold or more, was verified by Western blot analysis. In addition, protein levels of Cav1.2 and NCX1, which are abundant in cardiac muscle and showed negligible change in mRNA level in DCM, were also determined. Fig. 5 shows typical data and comparison of the averaged values of each protein level. Kv4.2 and KChIP2 were significantly decreased in DCM LV to less than 50% of values in the WT. Kv1.5 was also significantly decreased to 65% of WT. There was no significant difference in Kir3.1 between WT and DCM in spite of the significant difference in mRNA level. This is consistent with no significant change in K_Ach_ currents determined in isolated single myocytes (data not shown). There were also no significant differences in expression levels for Cav1.2 and NCX1 between WT and DCM groups. In summary, the decreases in Kv4.2, KChIP2 and Kv1.5 were significant in DCM and well explain the changes in *I_t_*_o_ and *I_Kur_*.

### Gene expression analysis in RV

**Figure 6:**
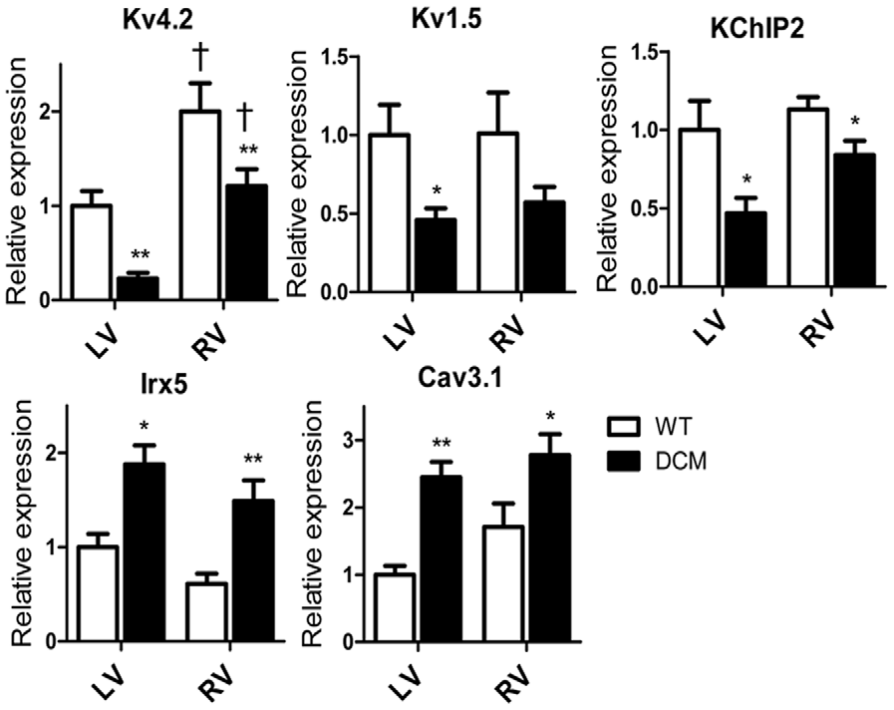
Comparison of mRNA expression between LV and RV. The expression levels of each gene are expressed relative to the average of WT LV. Data from LV are the same as in Figure 4. (n = 7 for each). **P<0.01, *P<0.05 between WT and DCM. †P<0.05 between LV and RV.

Gene expression was also examined in RV because they showed much less spontaneous activity than LV. Fig. 6 compares the expression levels of genes in which significant differences were detected between WT and DCM LVs (Fig. 4). In WT the level of Kv4.2 was 2-fold higher in RV than in LV, whereas 5-fold more abundant in DCM. Therefore, in DCM, the absolute amount of Kv4.2 mRNA in RV was not as low as that in LV. In addition, the decrements in KChIP2 was less marked in RVs than LVs. The expression levels of Kv1.5, Irx5 and Cav3.1 were similarly changed in DCM LV and RV. Thus the higher expression of the Kv4.2 and its accessory subunit, which is due to intrinsic property of RV and lesser down-regulation, may account for the shorter APD and less automaticity in DCM RV.

### Comparison of automaticity, APD_50_ and mRNA expression between 1-, 2- and 3-months old mice

**Figure 7:**
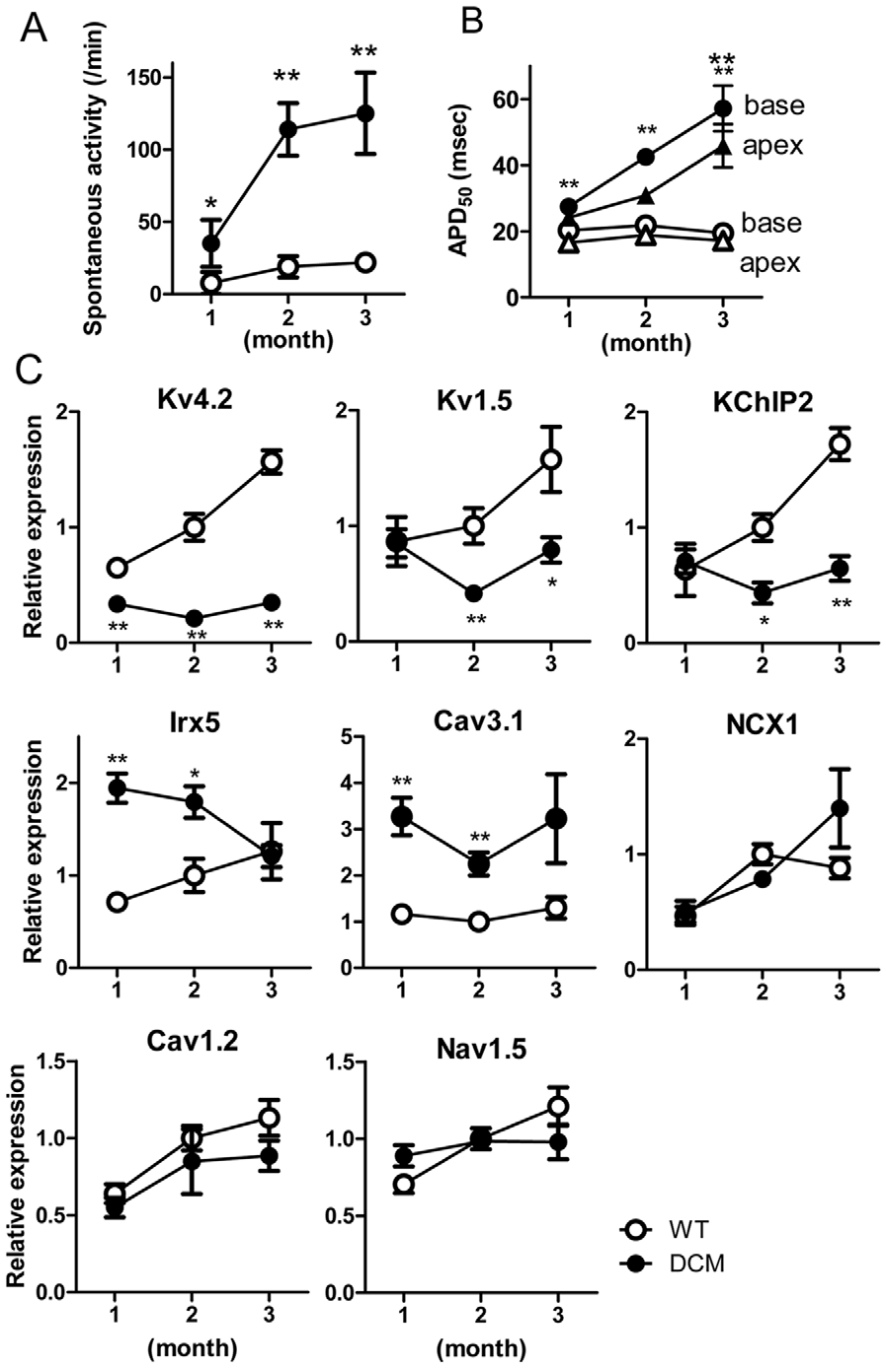
Spontaneous activities, APD_50_ and mRNA expression in LV from 1-, 2- and 3-month WT and DCM mice. **A**. Frequencies of spontaneous contractions after 3 Hz field stimulation in normal Krebs solution. (n = 9–14) **B**. APD_50_ values in LVs from mice at 1, 2 and 3 months (data are averages of 7–14 hearts). ○▵: WT; •▴: DCM. **C**. Comparison of mRNA expression in LVs at 1- (n = 5) 2- (n = 7) and 3-months (n = 7). The expression levels of each gene are expressed relative to the average in WT LV at 2 month. Data are means ± SEM. *P<0.05, **P<0.01 between WT and DCM at the same age.

Kaplan-Meier survival curves in Fig. 1A for these DCM model mice show that over 90% of the animals survive the first month. After 40 days, the survival rate decreases (also see [14]). The symptoms of HF emerge around 3 months (Fig. 1). To explore the changes occurring in DCM hearts during the 3 months after birth, functional parameters and expression levels of affected ion channel genes were determined. In DCM LVs from 1-month mice, spontaneous activity was detected, but its frequency was obviously less than at 2 months (Fig. 7A). In the WT LV, there were only small differences in APD_50_ values between 1- and 2-month mice (Fig. 7B). By contrast, APD_50_ in the DCM LV markedly increased from 1 to 2 months. The increments were more prominent at 3 months.

To determine which ion channels contribute to the age-dependent changes in automaticity and APD_50_ seen in DCM mice, we analyzed the expression levels of genes in which significant differences were detected between WT and DCM mice at 2 months (Fig. 7C). In LVs from 1-month DCM mice, the level of Kv4.2 expression was about 60% of that in WT mice and declined further to about 25% of WT at 2 months and later. Interestingly, Kv4.2 in the ventricle of the DCM neonate was already significantly decreased to 72±4% of the WT neonate (n = 6 for each). The levels of Kv1.5 and KChIP2 expression in DCM mice were similar to those in WT mice at 1 month, but they were significantly reduced at 2 months and later. In contrast, there were no significant differences in Cav1.2 and Nav1.5 between WT and DCM at 1–3 months. The expression of Cav3.1 was consistently higher in DCM mice than WT mice at 1 to 3 months. For NCX1, there was no difference between WT and DCM at 1 and 2 months, although it tended to increase at 3 months but without significant difference. The level of Irx5 in DCM was highest at 1 month and gradually decreased to the level of WT at 3 months, suggesting that some factor other than Irx5 may regulate the transcription of Kv4.2 in older DCM mice. In summary, among gene expression of molecules determined in this study, significant changes in Kv4.2, Irx5 and Cav3.1 started at 1 month or before, whereas changes in Kv1.5 and KChIP2 started at around 2 months.

## Discussion

In this study, we investigated how electrical remodeling and HF proceed in a knock-in mouse model of inherited DCM, in which myofibrillar Ca^2+^ sensitivity was intrinsically reduced [3], [14], [35]. This study indicates that progressive, multiple and qualitatively different types of electrical remodeling develop in DCM hearts prior to symptoms of HF.

### Time course of electrical remodeling and HF in DCM model

There are at least 3 stages in terms of development of HF and electrical remodeling in this DCM model. In stage 1, which is represented by DCM mice at 1 month, mortality was low with no clear symptoms of HF in spite of enlarged hearts. Slight prolongation of APD_50_ and moderate increase in spontaneous activity were detected in the myocardium. Gene expression of Kv4.2 was significantly lower than WT. Because the decrease in Kv4.2 and enlargement of the heart were already noticeable in neonates, these changes seem to occur during embryonic development. By contrast to Kv4.2, down-regulation of Kv1.5 or KChIP2 was not observed. The DCM model mice, at 1 month or before, are relatively safe with only modest electrical remodeling and no congestive HF.

In stage 2, which is represented by DCM mice at 2 months, they displayed high mortality without clear symptoms of HF. Their myocardium showed much more frequent spontaneous activity and further prolonged APD_50_ with an increased spatial difference compared to 1-month mice. These changes are associated with a reduction in K^+^ current density (*I_to_*, *I_Kur_*), which arises from substantial decreases in expression of Kv4.2, Kv1.5 and KChIP2, and may be related to the SD at around 2 months. Because knockdown of Kv4.2, or Kv1.5 alone does not result in SD or HF [36]–[37], combined decreases in Kv4.2, Kv1.5 and KChIP2 may play an important role in the frequent premature SD in DCM mice. As HF starts to develop after this stage, the DCM hearts at 2 months could maintain pump function in compensation for the increased electrophysiological vulnerability due to the remodeling of these ion channels.

In stage 3, represented by DCM mice at 3 months, DCM hearts enlarge further as many of them enter the true HF stage associated with lung edema. Expression levels of Kv4.2, Kv1.5 and KChIP2 are further decreased and APD_50_ is further prolonged. The expression of NCX1 tends to increase but without significant difference. Mice at this age may die with variable pathologies including lethal arrhythmias, which are related to severe HF.

The above time-course demonstrate that electrical remodeling precedes HF in this mouse model of human DCM mutation of the *TNTT2* gene. This time-course is consistent with the observations in human patients in which SD often occurs before awareness of the disease [2], [18]. In the human, however, onset and severity of symptoms are much more variable presumably due to various genetic and environmental factors. Detailed analysis using this animal model can help to reveal the time course of symptoms and electrical remodeling in this inherited DCM.

### Causes for prolongation of APD, spontaneous activity and arrhythmias

Our measurements showed that DCM cells at 2 months had a broader AP overshoot than WT cells. Among the three major repolarizing current components in mouse ventricular cells, *I_to_*, *I_Kur_*, and *I_ss_* [27], a prominent reduction was observed for *I_to_* and *I_Kur_*, while *I_ss_* was maintained at a comparable amplitude in DCM cells. In support of this, molecular correlates of *I_to_* and *I_Kur_* had a reduced expression in DCM cells at both mRNA and protein levels. Furthermore, these changes were less pronounced in RV at 2 months and LV in 1 month mice. These findings indicate that the prolonged APD in DCM cells is mostly attributable to the reduction of *I_to_* and *I_Kur_*.

The reduction of *I_to_* and *I_Kur_* in DCM cells, on the other hand, leads to a reduced repolarization reserve [38]. Since these current components are activated in the depolarized voltage-range (above −40 mV), their reduction in DCM cells would lead to a narrower margin for the occurrence EAD, thus providing an arrhythmogenic substrate. This notion is consistent with the actual occurrence of EAD in DCM cells (Fig. 2B).

The DAD was also observed in DCM cells. The prolongation of the APD may increase Ca^2+^ influx, enhance the Ca^2+^-extruding NCX reaction that provide more inward current, and cause depolarization at later stages of repolarization [39]–[40]. The β-adrenergic/cAMP system must also be considered as a source of DAD in DCM mice. This potentially causes an increased automaticity, especially *in situ*, by increasing the cAMP level to enhance phosphorylation of phospholamban and RyR2 and/or activate *I_f_* currents [20], [41]–[43]. Sympathetic tone has been reported to be elevated in these animals to maintain sufficient cardiac output whereas vagal activity is decreased [14], [20]–[21]. Consistently, more frequent arrhythmias were observed in DCM mice under mild restraint condition. Up-regulation of Cav3.1 raises a possible contribution of T-type Ca^2+^ currents in the development of myocardial automaticity in DCM mice, although our results showed no clear functional correlation. To summarize, the increased DAD and EAD may well explain the occurrence of premature ventricular complex (PVC) (Fig. 1) and ventricular tachycardia [14].

In addition to the increased spontaneous activity, myocardial fibrosis, which has been observed in 2 month mice[14], [44], and an increased spatial difference in APD (Fig. 2F) may also have some additional contribution to the lethal arrhythmias [45]–[46]. Both factors by themselves cannot cause spontaneous activity or PVC after T wave. However, myocardial fibrosis may contribute to sustain arrhythmia by a re-entry mechanism when re-entrant activity comes back at the repolarizing phase of action potential (R-on-T phenomenon). Also fibrosis and the increased spatial difference can facilitate dispersion of repolarization and T wave alternans [46] to worsen Torsades de pointes. Probably these factors in addition to the ion channel remodeling combine in development of severe arrhythmia. Further studies are required to elucidate the detailed developmental mechanisms leading to lethal arrhythmia in DCM.

### Comparison with other HF and DCM models

It is well known that HF and its preceding hypertrophic stage are also associated with electrical remodeling [39], [47]–[49]. Probably the electrical remodeling in this DCM model is, in part, related to those in HF [14]; however, there are distinctions. The most common ion channel remodeling in failing heart is reduction in *I_to_* and Kv4.2 transcription along with APD prolongation [10], [39], [47], and concurrent up-regulation of NCX1 [39], [47]–[49]. Changes in other K current components, including Kv1.5 and KChIP2, have been reported in some but not all cases [11], [39], [47]. In our DCM model, a decrease in Kv4.2 is already observed in neonates when HF is not evident. In contrast, a significant increase in NCX1 was not detected at 2 months. The time course and underlying mechanisms of electrical remodeling are not the same among the various types of HF and remain to be elucidated for each HF model.

Probably the mutation in *TNNT2* is not directly and specifically responsible for the arrhythmia because similar lethal arrhythmias are also frequently found in DCM patients with gene mutations of other proteins [1]–[4]. The DCM heart presumably undergoes a complex series of changes in neurohumoral mechanisms such as the sympathetic nervous (SNS) and renin–angiotensin–aldosterone systems (RAAS) to compensate for the reduction in cardiac contractility [39]. These mechanisms are likely to affect cardiac gene expression resulting in the electrical remodeling. Interestingly, among mouse models that develop characteristics of DCM, an electrical remodeling similar to our model has been reported in the transgenic mouse model of α_1B_-adrenergic receptor overexpression (α_1B_-AR mice), in which a phenotype mimics idiopathic DCM and results in HF and/or death with arrhythmia [11]. In this α_1B_-AR mouse model, the down-regulation of Kv4.2, Kv1.5 and KChIP2 was evident at a young age (2–3 months) before development of HF (9–12 months), although onset-timing of those changes is unclear. The similarity between their and our model raises an interesting possibility that activation of the SNS involving α_1B_-AR system is related to the remodeling in our ΔK210 mice. The next step will be to clarify what kinds of factors cause the multiple types electrical remodeling. At least three major factors may involve these multistep remodeling.

### Conclusions

Our results indicate that multiple types of progressive electrical remodeling occur at different time points in the hearts of DCM model mice, and that combined electrical remodeling in DCM model mice prolongs APD and increases the excitability of ventricular myocytes, which can develop ventricular arrhythmia. To increase our understandings of the mechanisms of SD in inherited DCM in general, further studies with other knock-in models are absolutely required. Because the DCM mice survive relatively well while the extent of electrical remodeling remains small, early initiation of therapy to suppress remodeling may be a key to prevent SD in inherited DCM.

## Materials and Methods

### Animal model

All experiments were carried out in accordance with the Ethics Committee guidelines and were approved by the Committee for Animal Experimentation of Juntendo University (approval number 230021) and by Saga University Animal Care and Use Committee. The investigation conforms to *Guiding Principles for the Care and Use of Animals in the Field of Physiological Sciences* (Physiological Society of Japan).

A knock-in mouse with deletion mutation, Lys-210, in its endogenous cardiac troponin T gene (*Tnnt2* ΔK210) was used as the DCM model animal [14]. These mice had been backcrossed to the C57BL/6J line for at least 10 generations and were maintained under specific pathogen-free (SPF) conditions. Mixed-gender homozygous mutant and wild type (WT) mice were obtained by crossing heterozygous mutant mice and were used as DCM and non-DCM models, respectively.

### Electrocardiography

Electrocardiography (ECG) lead II was recorded from mice anesthetized with an intraperitoneal injection (i.p.) of pentobarbital (20–25 mg/kg) using ECG Amplifier (Nihon Koden, Japan). ECG records of conscious mice were obtained with ECGenie (Mouse Specifics, Inc. MA, USA), which took signals noninvasively from palms and planters of animals using footplate electrodes. To determine RR, QRS and QT intervals, ECG signals were recorded for 2 min for each mouse. To detect arrhythmia in mice, they were placed on the footplate electrodes for 30 min in an oval plastic dome with openings at mouth and tail (7 cm length, 4 cm width, and 4 cm height) to restrict free movement. This procedure gave mice mild restraint stress to increase frequency of arrhythmias and allowed us to analyze many mice repeatedly. Records were analyzed with ECG Analysis software (AD Instruments, Japan).

### Determination of wheel running activity

To estimate the extent of heart failure of DCM mice, physical activity was evaluated by measuring voluntary wheel running activity [22] (paper in preparation). WT and DCM mice at 6 weeks or later were housed with free access to a running wheel (6 cm radius, Mini Mitter Co. Inc. OR, USA) for two days a week and their running activities were measured (round/day). Running activities at 2 and 3 months were determined from the same animals.

### Solutions and reagents used in experiments with myocardium

Normal Krebs solution for myocardium experiments contained (mM): 120 NaCl, 5 KCl, 25 NaHCO_3_, 1 NaH_2_PO_4_, 2 CaCl_2_, 1 MgCl_2_, 10 glucose and was saturated with 95% O_2_-5% CO_2_. High-K^+^ Krebs solution used for muscle preparation contained 25 mM KCl instead of 5 mM. Di-4-ANEPPS was obtained from Invitrogen/Molecular Probes (Eugene, OR, USA).

### Experiments with isolated myocardium

Mice at 1, 2 and 3 months of age were deeply anesthetized with pentobarbital sodium (100 mg/kg i.p.) and heparin (100 unit/kg). The hearts and lungs were excised, rinsed in Krebs solution and their weights measured. The hearts were perfused via the aorta with a high-K^+^ Krebs solution. Muscles of the left ventricles (LV) were dissected in high K^+^ solution and then kept in normal Krebs solution at room temperature. Measurements were made 1 to 3 h after preparation.

Membrane potential imaging experiments were carried out as described previously with some modification [23], [50]–[51]. LV muscles were loaded with 25 µM di-4-ANEPPS for 20 min, after which they were mounted in a chamber on the stage of an inverted microscope equipped with the Nipkow disc confocal system (CSU22, Yokogawa, Japan) and a W-view system (Model 8509, Hamamatsu Photonics, Hamamatsu, Japan). Di-4-ANEPPS was excited by 488 nm laser light and fluorescence images at 525 and 620 nm were simultaneously captured, side-by-side, using the same camera, and ratio images were then calculated. To determine the optical AP duration (APD), membrane potential signals were obtained at 3.67 ms intervals as 16×32 pixel signals (binning = 16×16) from 160×320 µm areas. The APD_50_ (i.e., the time at which the down-stroke of the AP had recovered 50% toward baseline) was obtained as the activation minus the repolarization time points. To monitor AP signals for longer periods, 2-dimensional images were obtained at 8.7 ms intervals (64×128 pixels with binning = 2×2). Experiments were carried out at 25∼27°C.

To detect spontaneous activity of myocardium, isometric contractions were also determined. Papillary muscles were isolated from the LV (0.5–1.0 mm in diameter) and mounted horizontally, using silk thread, between a force transducer and a fixed hook in a chamber. They were perfused with Krebs solution at 30°C at a rate of 2 ml/min and electrically field-stimulated at 0.5 or 3 Hz using a suprathreshold voltage. To evaluate automaticity, spontaneous contractions were recorded for 1 min in the absence of stimulation following conditioning pulses applied at 3 Hz for 15 min.

### Whole-cell clamp recording

Single heart cells were isolated from left ventricles of mice using an established enzymatic method [52]. The mice were killed by an overdose injection of pentobarbital sodium (300 mg/kg, i.p.). After isolation, the cells were whole-cell clamped in the current- or voltage-clamp mode using a patch-clamp amplifier (CEZ-2300, Nihon Koden). Patch-pipettes (2–3 MΩ) were pulled from thin-wall glass capillaries (14-084-12, Hilgenberg), and series resistance (3–5 MΩ) was compensated by 50–70% electronically. Data acquisition and analysis were done using pClamp 10 software suit and a DigiData 1440A signal interface (Molecular Devices). All voltage data were corrected for a liquid junction potential of −8 mV assumed at the pipette tip.

Whole-cell clamp records were acquired at 37°C. For recording APs, the cells were stimulated at 5 Hz by injecting 5 ms supra-threshold current pulses via the patch-pipette. They were superfused with Tyrode solution containing (mM): 140 NaCl, 5.4 KCl, 1.8 CaCl_2_, 0.5 MgCl_2_, 0.33 NaH_2_PO_4_, 11 glucose, and 5 HEPES-NaOH (pH 7.4). Pipette solution contained (mM): 110 K-aspartate, 30 KCl, 10 NaCl, 5 Mg-ATP, 0.1 Tris-GTP, and 20 HEPES-KOH (pH 7.2). For recording the membrane current, 1 s command pulses were applied to the cells every 4 s from a holding potential of −78 mV. To eliminate L-type Ca-current and [Ca]_i_-activated currents, Tyrode solution was modified to contain 0.1 mM CdCl_2_, as well as the pipette solution to contain 4 mM BAPTA and no NaCl. Detailed procedures for separation of *I_to_*, and *I_Kur_* are described in Method S1.

### Real-time RT-PCR

Total RNA was isolated from the LV using an RNeasy Fibrous Tissue Mini Kit (Qiagen) and treated with DNase I to prevent contamination with genomic DNA. First strand cDNA was synthesized using High-Capacity RNA-to-cDNA Master Mix (Applied Biosystems). The expression of genes encoding Kv1.5, Kv2.1, Kv4.2, Kir2.1, Kir2.2, Irx5, KChIP2, Nav1.5, Cav1.2, Cav3.1 and NCX1 was assessed using quantitative real-time PCR analysis. The oligonucleotide primers used for the real-time PCR are listed in Table S1. PCR was carried out using ABI PRISM 7500 Real Time PCR Systems. The cycling protocol included an initial stage at 95°C for 10 min, followed by 40 cycles at 95°C for 15 s and 60°C for 1 min. To ensure the validity of the result, the linearity and the efficiency criteria (slope of −3.1 to −3.6 in the C_t_ vs log-template amount) were carefully followed. The mRNA expression level of each gene in each sample was quantified relative to that of the Glyceraldehyde 3-phosphate dehydrogenase (GAPDH) gene in the same sample. For each gene, data from individual samples were normalized to the average values from LV of 2-month-old WT mice.

### Western blot analysis

Western blotting was carried out with membrane protein samples. Briefly, LVs (10–20 mg) were dissected from WT and DCM mice and homogenized in a buffer containing 0.3 M sucrose, 20 mM MOPS, pH 7.4 and a cocktail of protease inhibitor (aprotinin, antipain, chymostatin, leupeptin, and pepstatin A at 2 µg/ml each). The homogenate was centrifuged for 10 min at 2,000 g to remove contractile proteins and insoluble materials. The supernatant was centrifuged for 20 min at 100,000 g to sediment the membrane fractions. The resultant pellet was washed twice with the buffer and resuspended in a small volume (50 µl) of the buffer. Protein concentrations were determined by Advanced protein assay kit (Cytoskeleton Inc.). The protein samples (50 µg protein for each) were separated by SDS-PAGE with a 3–15% gradient gel and then transferred onto a polyvinylidene fluoride membrane. The membrane was blocked with 5% nonfat dry milk powder in TBS (150 mM NaCl, 20 mM Tris-Cl, pH 7.5) containing 0.05% Tween 20 (TTBS) for 1 h at room temperature and probed with primary antibodies overnight at 4°C. The antibodies used are: rabbit polyclonal anti-Kv4.2 (Alomone labs, APC-023), mouse monoclonal anti-KChIP2 (UC Davis/NIH NeuroMab Facility, clone K60/73), goat polyclonal anti-Kv1.5 (Santa Cruz Biotechnology, sc-11679), rabbit polyclonal anti-Cav1.2 (Alomone labs, ACC-003), mouse monoclonal anti-NCX1 (Abcam, ab6495), and mouse monoclonal anti-β-actin (Abcam, ab8226). After three washings with TTBS, the membrane was then probed with horseradish peroxidase-conjugated secondary antibodies for 2 h at room temperature. After extensive washings with TTBS, the positive bands were detected by LAS3000 lumino image analyzer (Fujifilm, Japan) using ImmunoStar LD chemiluminescence detection reagents (Wako Pure Chemical Industries). For polyclonal antibodies, validations of the specific bands were confirmed with the antigen peptides provided by the manufacturers. The densities of specific bands were determined using MultiGauge software (Fujifilm, Japan) and corrected for the density of β-actin.

### Statistics

Data are presented as means ± SEM. Statistical comparisons have been made using GraphPad Prism5 Software (USA). Student's *t*-test was used to compare means of two groups. P values<0.05 were considered significant.

## Supporting Information

Method S1 Procedures for isolation of *I_to_* and *I_Kur_*.
(DOC)

Table S1 Real-time PCR primer sequences.
(DOC)
