# Supplementary material for: Multistep Ion Channel Remodeling and Lethal Arrhythmia Precede Heart Failure in a Mouse Model of Inherited Dilated Cardiomyopathy
Source: PLoS One. 2012 Apr 13;7(4):e35353. doi: 10.1371/journal.pone.0035353 (PMC3325934; doi:10.1371/journal.pone.0035353)
Supplement: Method S1 — Procedures for isolation of Ito and IKur. (DOC) [file pone.0035353.s001.doc]

**Method S1**

**Isolation of *I_to_* and *I_Kur_***

The depolarization-activated outward whole-cell current involved two time-dependent current components: *I_to_* and *I_Kur_* [1]. As illustrated in Fig. S1A, the slow component *I_Kur_* (τ = around 150 ms) was sensitive to 100 μM-4AP, which did not affect the fast component *I_to_* (τ= around 10 ms). Meanwhile, the *I_to_* was sensitive to an inactivating prepulse (20 ms to -8 mV), which did not affect the *I_Kur_*. Taking advantage of these properties, the *I_to_* was isolated as the current component sensitive to the inactivating prepulse but not to 100 μM-4AP (Fig.S1B). The *I_Kur_* was isolated as the current component sensitive to 100 μM-4AP but not to the inactivating prepulse (Fig. S1C).

**References**

[1] Brouillette J, Clark RB, Giles WR, Fiset C. Functional properties of K^+^ currents in adult mouse ventricular myocytes. J Physiol. 2004;559:777-98.

Fig. S1


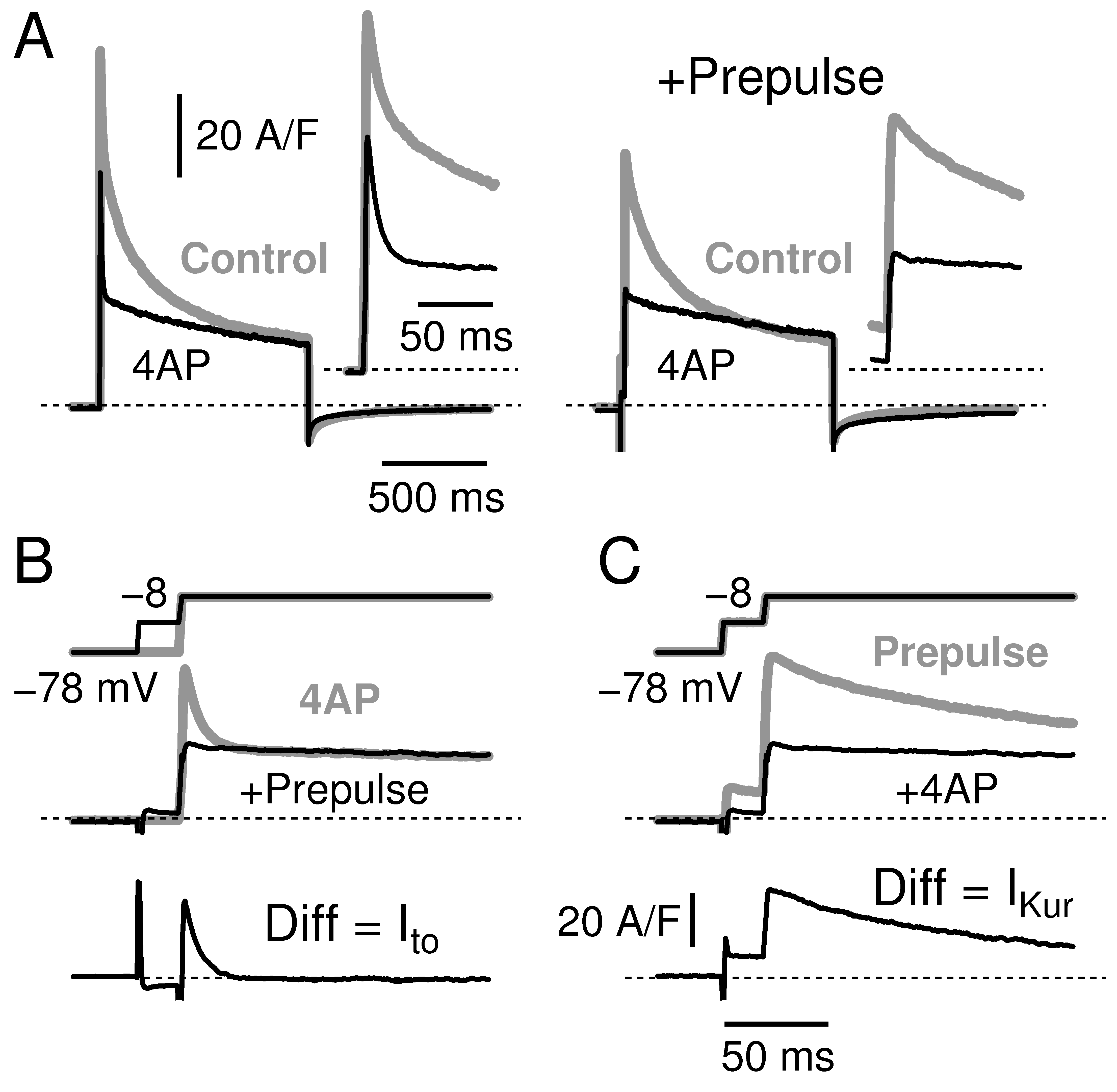


**Figure S1. Isolation of *I_to_* and *I_Kur_*.** (**A**) The effects of 100 μM-4AP and an inactivating prepulse on the outward whole-cell current. The records were acquired using depolarizing pulses (1 s to +52 mV), with (right) or without (left) an inactivating prepulse (20 ms to -8 mV), from a holding potential of -78 mV. Records acquired with (black) and without 100 μM-4AP (gray) are shown superimposed. Insets expand each record in a faster time-base. (**B**) For isolating *I_to_*, current records (upper) were acquired using 100 μM-4AP, with (black) or without (gray) using the inactivating prepulse (top). Their difference provided the *I_to_* (bottom). (**C**) For isolating *I_Kur_*, current records were acquired using the inactivating prepulse (top), with (black) or without (gray) using 100 μM-4AP (middle). Their difference provided the *I_Kur_* (bottom). Dotted lines indicate zero-current level.
