# Supplementary material for: Multistep Ion Channel Remodeling and Lethal Arrhythmia Precede Heart Failure in a Mouse Model of Inherited Dilated Cardiomyopathy
Source: PLoS One. 2012 Apr 13;7(4):e35353. doi: 10.1371/journal.pone.0035353 (PMC3325934; doi:10.1371/journal.pone.0035353)
Supplement: Table S1 — Real-time PCR primer sequences. (DOC) [file pone.0035353.s002.doc]

**Table S1** Real-time PCR primer sequences

| Target  (bp) | Accession | Forward primer | Reverse primer |
| --- | --- | --- | --- |
| GAPDH  (124) | NM_008084 | AACTTTGGCATTGTGGAAGG | GGATGATGTTCTGGGCAGC |
| Kv4.2  (126) | NM_019697 | GCAAGCGGAATGGGCTAC | TGGTTTTCTCCAGGCAGTG |
| Kv2.1  (132) | NM_008420 | ACCTGTCTCCCAACAAGTGG | CGTTCAAGTGCTGCGGA |
| Kv1.5  (123) | NM_145983 | TCAAGGAAGAGGAGAAGCCC | GAATGACCAAGACCGACACG |
| KChIP2  (112) | NM_030716 | AACTATCCACGGTGTGCCAC | GGACATTCGTTCTTGAAGCCT |
| Irx5  (74) | NM_018826 | ATCCTCGGTCATCCACTCG | ACCACAGTTTGGGCTTGGC |
| Kir2.1  (186) | NM_008425.2 | TGAAGTTGCCCTAACAAGCA | AAAGTAAGTATGACAAAGACGGAA |
| Kir2.2  (167) | NM_010603.3 | CGCCAACTCTTTCTGCTATG | ATCTCCGACTCCCGTCTGT |
| Kir3.1  (127) | NM_008426 | CTTCTATGACCTATCCCAGCG | CACTTCGTCCTCTGTGTATGATG |
| Nav1.5  (149) | NM_021544 | GCAGAAGGTGAAGTTCGTGG | TGAAGACCAAGTTTCCGACC |
| Cav1.2  (98) | NM_009781 | CCCTTCTTGTGCTCTTCGTC | TATGCCCTCCTGGTTGTAGC |
| Cav3.1  (101) | NM_009783 | TCCTTGGAGATGCTGCTGAAG | CACACGCTGATGACCACAATG |
| NCX1  (84) | AF_004666 | GAGGTTGGAGCAGTTGGAAG | ACCAGACGAAATCCCATTGA |
